# Effect of Xuezhikang Therapy on Expression of Pulmonary Hypertension Related miR-638 in Patients With Low HDL-C Levels

**DOI:** 10.3389/fphar.2021.764046

**Published:** 2021-12-20

**Authors:** Ruihua Cao, Tao Sun, Ruyi Xu, Jin Zheng, Hao Wang, Xiaona Wang, Yongyi Bai, Ping Ye

**Affiliations:** ^1^ Department of Cardiology, The Second Medical Center and National Clinical Research Center for Geriatric Diseases, Chinese PLA General Hospital, Beijing, China; ^2^ Department of Cardiology, Huashan Hospital, Fudan University, Shanghai, China; ^3^ Beijing Key Laboratory of Precision Medicine for Chronic Heart Failure, Chinese PLA General Hospital, Beijing, China

**Keywords:** Xuezhikang, therapy, pulmonary hypertension, miR-638, low HDL-C

## Abstract

**Objective:** Low plasma level of high-density lipoprotein cholesterol (HDL-C) associated with poor outcomes in several cardiovascular diseases, including pulmonary arterial hypertension (PAH). Regulation of miR-638 have been proved to be associated with PAH. The aim of this study was to evaluate the expression of miR-638 after Xuezhikang (XZK) therapy in patients with low HDL-C.

**Methods:** Plasma levels of miR-638 were quantified by real-time polymerase chain reactions in 20 patients with PAH and 30 healthy controls. A total of 40 subjects with low HDL-C were assigned to receive an XZK therapy for 6 months. The miR-638 expression profiles were detected in PAH patients, XZK-treated subjects and lovastatin treated pulmonary arterial smooth muscle cells (PA-SMCs).

**Results:** The relative expression level of miR-638 in the plasma was lower in the PAH patients than that in the controls (*p* < 0.001). An increase of 11.2% from baseline in the HDL-C level was found after XZK therapy (*p* < 0.001). The relative expression of miR-638 was increased after XZK treatment (*p* < 0.01). The changes of miR-638 were inversely associated with baseline HDL-C levels. A significantly reduction in miR-638 expression were found in PDGF-BB-treated hPA-SMCs compared to the control cells, and the pre-treatment of the cells with lovastatin significantly re-gain the expression levels in miR-638.

**Conclusion:** In patients with low HDL-C levels, XZK therapy raised the expression of miR-638, suggesting that the potential therapeutic effect of XZK in PAH patients with low serum HDL-C levels deserves further exploration.

## Introduction

Pulmonary arterial hypertension (PAH) is a devastating and lethal cardio-pulmonary disease (Hoeper et al., 2016). Epidemiologic studies have demonstrated that low plasma level of high-density lipoprotein cholesterol (HDL-C) was associated with poor outcomes in pulmonary arterial hypertension (Rayner et al., 2010). However, clinical trials indicated that raising the levels of HDL-C did not achieve the expected clinical benefit, which may be due to the change of particle size and the function of HDL-C (Keene et al., 2014; Probstfield et al., 2018). In view of the complex function of HDL, our understanding in the metabolic mechanisms of HDL is still incomplete.

MicroRNAs (miRNAs) are conserved small non-coding RNA molecules, as important posttranscriptional regulators of lipid metabolism, and important targets for therapeutic intervention (Poller et al., 2018; Sun et al., 2020). Circulating microRNAs (miRNAs) exist in body fluids in a stable, cell-free form, and are closely related to human diseases (Reddy et al., 2019). Several miRNAs have been demonstrated to regulate lipid metabolism, including miR-122, miR-370, miR-335, miR-378/378* and miR-33 (Rayner et al., 2010; Rayner et al., 2011a; Rayner et al., 2011b; Kim et al., 2017; Agbu and Carthew, 2021). Previous studies have demonstrated that MCT resulted in significant pulmonary vascular remodeling and down-regulation of miR-638; miR-638 mimics inhibited pulmonary arterial smooth muscle cells (PA-SMCs) proliferation and percentage of PCNA-positive cells *in vitro* (Luque et al., 2018; Liu et al., 2020; Mirhadi et al., 2021). The roles of miR-638 in lipid metabolism and expression profiles in patients with PAH are unclear.

Xuezhikang, an extract of cholestin, containing a combination of lovastatin, phytosterols and isoflavones. Each 1,200 mg XZK capsule contains about 10 mg lovastatin (Jia et al., 2016). It has the similar lipid-lowering effects with statins (Li et al., 2010). Our previous study demonstrated for the first time that plasma levels of miR-33a and miR-33b were significantly increased after XZK treatment, and the changes of miR-33a and miR-33b were inversely associated with the baseline levels of LDL-C (Cao et al., 2014). However, the potential mechanism of XZK in HDL-raising effect is still not completely clear. Furthermore, the effect of XZK therapy on the expression of miR-638 and whether there are “cross-talk” among pulmonary hypertension, miR-638 and HDL-C metabolism need further investigation.

In the present study, we aim to investigate relationship among the expression of miR-638, XZK treatment and pulmonary hypertension.

## Methods

### Samples Obtained From Pulmonary Arterial Hypertension Patients

Blood samples were obtained from PAH patients and healthy controls for the PAH case-control study. Twenty patients with PAH were consecutively enrolled from January 2019 to December 2019 at the Chinese PLA General Hospital and Huashan Hospital. The clinical diagnosis of PAH was according to the 2015 ESC/ERS guidelines for the diagnosis and treatment of pulmonary hypertension (Galiè et al., 20152016). Thirty normal volunteers matched for age, gender and race were enrolled simultaneously. All blood samples were centrifuged at 3,000 ×g for 10 min at room temperature, and the obtained serum samples were stored at −80°C for RNA extraction.

### Subjects of Xuezhikang Study

From September 2010 to June 2011, patients were screened and enrolled from a community in the Pingguoyuan area of the Shijingshan district, Beijing, China. After signing informed written consent, 150 participants were tested for low plasma HDL-C levels. Eighteen subjects with bedridden status, severe systemic diseases or mental illness were excluded.

Clinical data collection and biomarker variable determination were obtained from the 132 subjects. Among them, 42 patients with low HDL-C levels <40 mg/dl (1.03 mmol/L) for men; <50 mg/dl (1.29 mmol/L) for women) were eligible for analysis (Galiè et al., 20152016). Patients who have taken any lipid-lowering medication within the past 4 weeks required to discontinue lipid-modifying drugs before registration, in order to obtain accurate baseline blood lipid values. All patients who were able to adapt during the study were assigned to take an XZK capsule, 600 mg twice daily (Beijing WBL Peking University Biotech Co., Ltd., Beijing, China) for 6 months.

The study was approved by the ethics committee of the Chinese PLA General Hospital, and each participant provided with written informed consent.

### Laboratory Measurements

Blood samples were centrifuged immediately and stored at −80°C. Concentrations of fasting glucose, total cholesterol (TC), triglyceride (TG), HDL-C, LDL-C and creatinine were determined using the Roche enzymatic assays (Roche Diagnostics GmbH, Mannheim, Germany) on a Roche autoanalyser (Roche Diagnostics, Indianapolis, IN, United States).

### Cell Culture Experiments

Human PA-SMCs (CP3110) and smooth muscle cell medium were purchased from ScienCell Research Laboratory (Calsbad, CA, United States). The hPA-SMCs were incubated in smooth muscle cell medium, which contained 20% fetal bovine serum and 1% penicillin and streptomycin and were cultured at 37°C with 5% CO_2_ in humidified conditions.

The hPA-SMCs were seeded in Costar six 6-plate at a concentration of 2 × 10^5^/well. After the cells were 80% confluent, they were then starved in an FCS-free medium for next 24 h, followed by pre-treated with lovastatin (Sigma, MO, United States, at a final concentration of 1 or 5 μM/L) or equal volume control (1% dimethylsulfoxide), and were exposed to PDGF-BB (Thermo Fisher, MA, United States) at a concentration of 20 ng/ml for next 48 h. At the endpoint, the cell supernatant was removed and cells were lysized with Trizol for total RNA isolation.

### RNA Isolation and Real-Time Polymerase Chain Reaction

Total RNA from hPA-SMCs and plasma was isolated with the use of Trizol as previous described (Bai et al., 2021) and the extracted RNA was reverse transcribed in the presence of a poly-A polymerase with an oligo-dT adaptor. Real-time PCR was carried out with SYBR green detection using hsa-miR-638 and a universal adaptor reverse primer. Relative expression was evaluated by the comparative Ct (threshold cycle) method and normalized to the expression of U6 or U48 small RNA.

### Statistical Analysis

Continuous variables are expressed as mean ± standard deviation (SD) or median; whereas dichotomous variables are expressed as numbers and percentages. Data that followed a normal distribution and met variance homogeneity were analyzed for statistical significance by unpaired Student’s t test or ANOVA followed by Bonferroni’s multiple comparison post hoc test. When the data did not meet the variance homogeneity, one-way ANOVA followed by Dunnett’s multiple comparison post hoc test was used. The categorical variables were compared by chi-square test. Pearson correlation coefficients was used to analyzed the strength of the correlation between continuous variables. The analyses were performed using SPSS (version 17.0, Inc., Chicago, IL, United States). Statistical significance was set at *p* < 0.05, all reported *p* values are two-tailed.

## Results

### Baseline Characteristics of Subjects of Xuezhikang Study

Of the 42 enrolled patients, two were lost after 6 months of follow-up. Finally, 40 subjects (mean age 58.7, 41–77 years, eight males) were eligible for analysis. The baseline demographic characteristics of the 40 patients who completed the trial are summarized in Table 1.

**TABLE 1:** Baseline characteristics of Xuezhikang study.

Characteristics	*N* = 40
Age (years)	58.7 ± 10.6
Male gender, *n* (%)	10 (25)
Hypertension *n* (%)	24 (60)
BMI(kg/m^2^)	28.1 ± 4.8
SBP (mmHg)	138 ± 15
DBP (mmHg)	87 ± 9
TC (mmol/L)	5.54 ± 0.76
TG (mmol/L)	2.85 (2.16, 3.97)
HDL-C (mmol/L)	1.07 ± 0.13
LDL-C (mmol/L)	3.35 ± 0.72
Hcy (μmol/L)	14.6 ± 6.7

BMI, body mass index; SBP, systolic blood pressure; DBP, diastolic blood pressure; TC, total plasma cholesterol; HDL-C, high-density lipoprotein cholesterol; LDL-C, low-density lipoprotein cholesterol; Hcy, Homocysteine are given as mean ± standard deviation. Triglyceride (TG), values as median (quartile 1, quartile 3).

### Effects of Xuezhikang Treatment on Lipid Profiles

**FIGURE 1:**
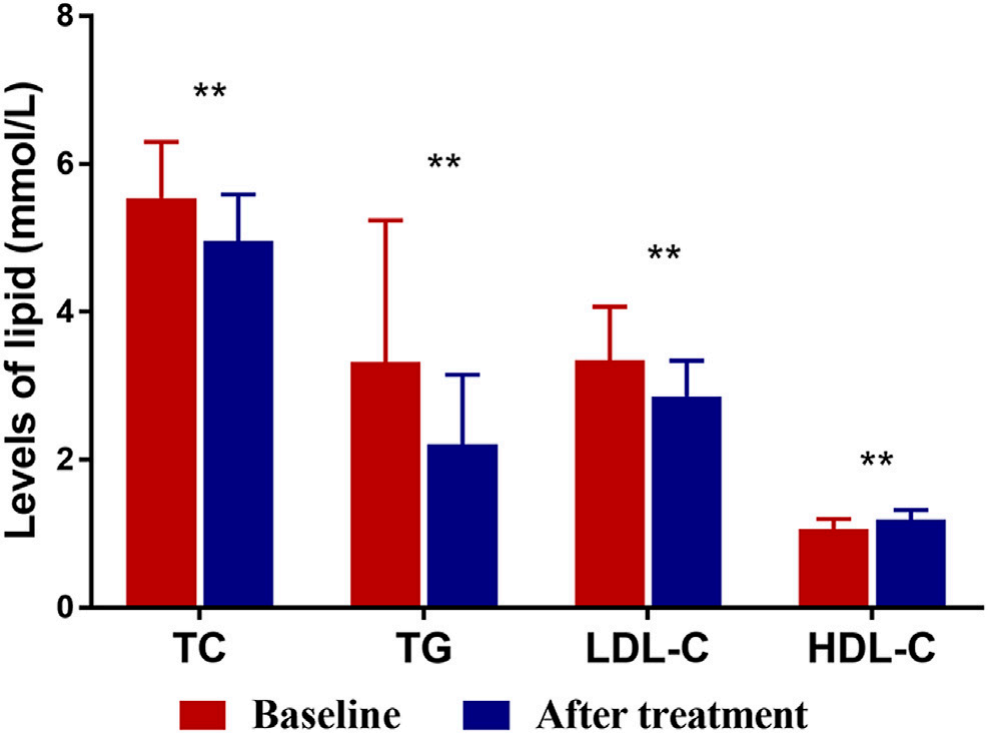
The levels of TC, TG, LDL-C and HDL-C at baseline and after XZK treatment. After XZK treatment, the levels of TG, TC and LDL-C decreased significantly, while the levels of HDL-C increased significantly (**p < 0.01). Horizontal lines represent standard deviation.

Figure 1 and Table 2 summarizes lipid profiles obtained in patients who completed the trial. The mean HDL-C level was 1.19 ± 0.13 mmol/L after XZK treatment, representing an increase of 11.2% from baseline (*p* < 0.001). The mean TG level after XZK treatment was 2.21 ± 0.94 mmol/L, a 22.5% reduction from baseline (*p* < 0.001). The mean LDL-C level was 2.86 ± 0.48 mmol/L after XZK treatment, representing a reduction of 14.6% from baseline (*p* < 0.001).

**TABLE 2:** Changes of lipid profiles after Xuezhikang treatment.

Lipid profiles	Baseline	After treatment	Percent change (%)	*p* Value
TC (mmol/L)	5.54 ± 0.76	4.96 ± 0.63	−10.5	<0.001
TG (mmol/L)	3.32 ± 1.92	2.21 ± 0.94	−22.5	<0.001
LDL-C (mmol/L)	3.35 ± 0.72	2.86 ± 0.48	−14.6	<0.001
HDL-C (mmol/L)	1.07 ± 0.13	1.19 ± 0.13	11.2	<0.001
HDL-C/LDL-C ratio	0.33 ± 0.07	0.43 ± 0.07	30.3	<0.001

TC, total plasma cholesterol; HDL-C, high-density lipoprotein cholesterol; LDL-C, low-density lipoprotein cholesterol; TG, triglyceride.

### Effects of Xuezhikang Treatment on miR-638 Expression

**FIGURE 2:**
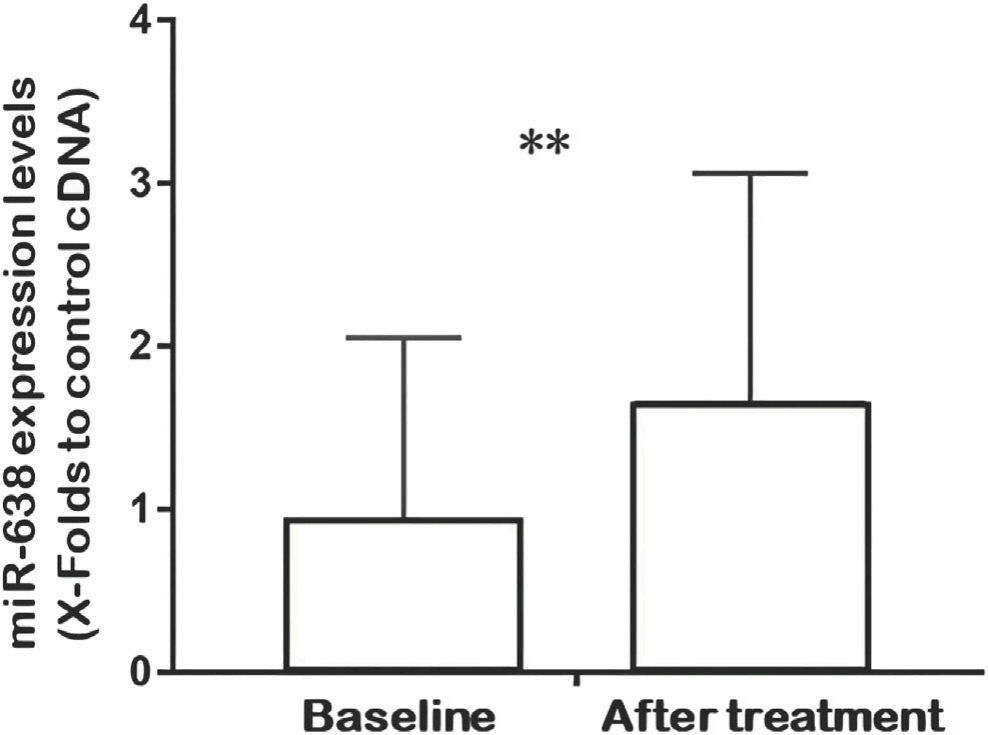
Quantitative real-time fluorescence polymerase chain reaction (QRT-PCR) analysis of miR-638 expression at baseline and after XZK treatment. Relative expression of miR-638 was raised after XZK treatment. **p < 0.01. Horizontal lines represent standard deviation.

**FIGURE 3:**
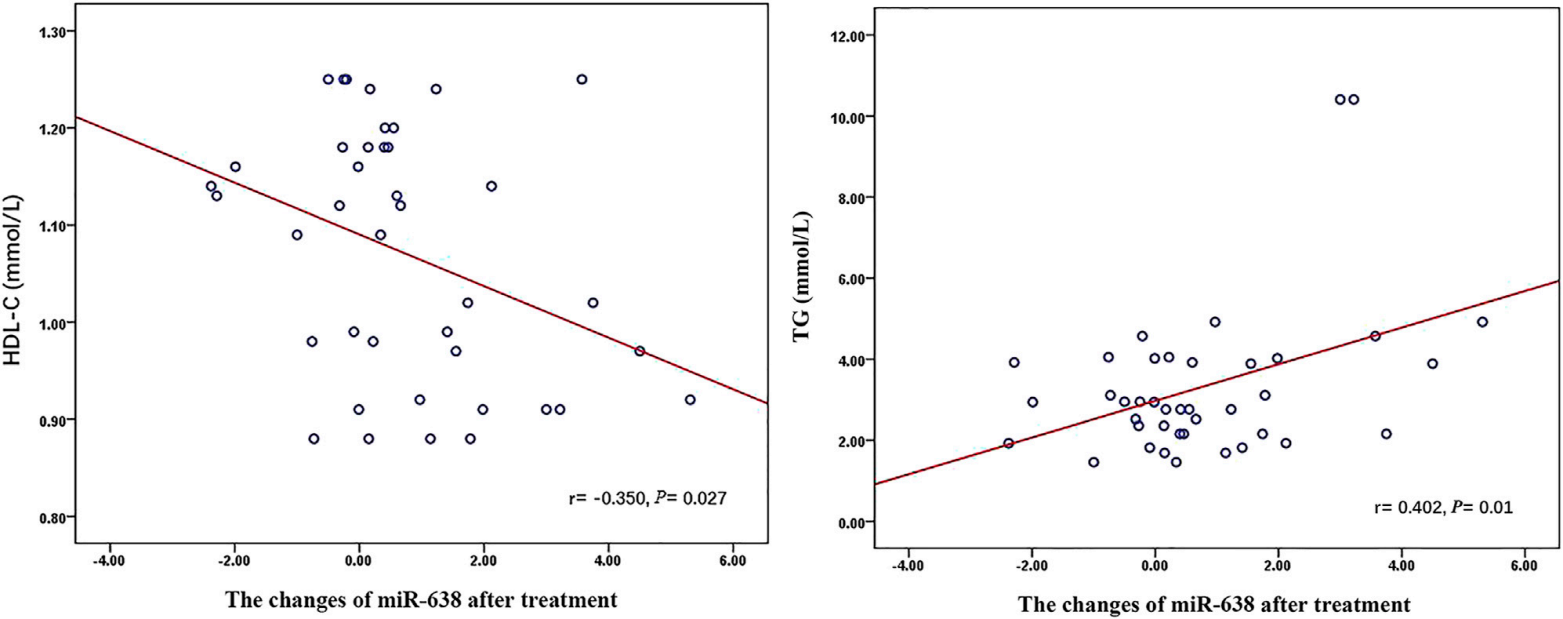
The relationship between miR-638 and Lipid Profiles after XZK treatment. The changes of miR-638 were negatively correlated with baseline HDL-C levels (*r* = −0.350, *p* = 0.027). Conversely, the changes of miR-638 correlated positively with baseline triglyceride (TG) levels (*r* = 0.402, *p* = 0.01).

Q-PCR analysis of plasma miRNAs showed that the relative expression of miR-638 increased after XZK treatment (*p* = 0.008) (Figure 2). The changes of miR-638 were negatively correlated with baseline HDL-C levels (*r* = −0.350, *p* = 0.027). Conversely, the changes of miR-638 correlated positively with baseline triglyceride (TG) levels (*r* = 0.402, *p* = 0.01) (Figure 3).

### Expression of miR-638 in Pulmonary Arterial Hypertension Patients and Lovastatin Treated PASMCs

**FIGURE 4:**
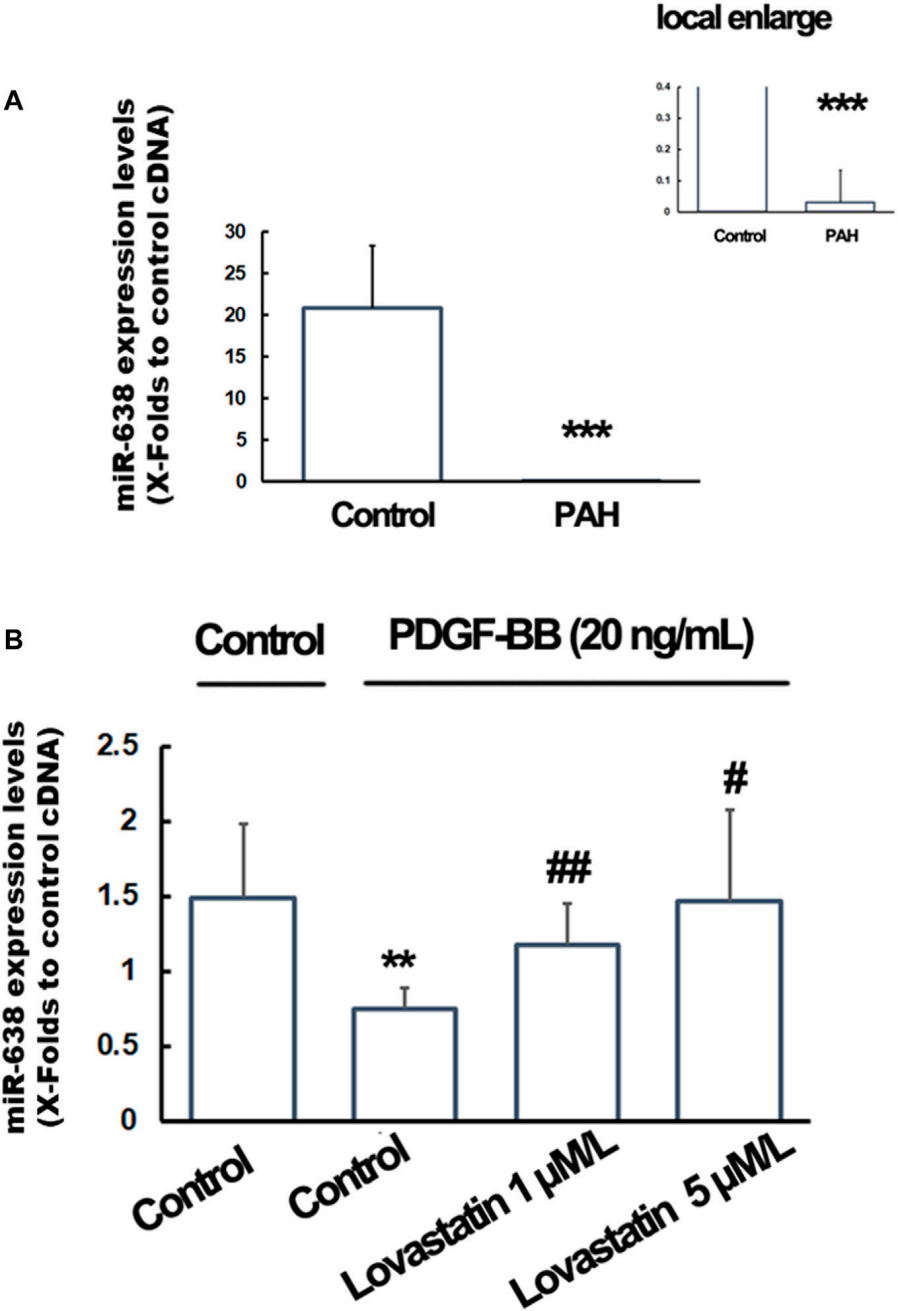
Expression of miR-638 in PAH patients and lovastatin treated PASMCs. Total RNA from the plasma of PAH patients and hPA-SMCs were isolated with the use of Trizol and the expression levels of miR-168 were detected using qRT-PCR. **(A)**. The plasma miR-638 levels were significantly decreased in PAH patients (*n* = 20) compared with controls individuals (*n* = 30). Data was expressed as Mean ± SD. *p < 0.05, **p < 0.01, ***p < 0.001 vs the normal control. U48 were used as a control gene. **(B)**. The starved human PA-SMC were pre-treated with lovastatin at a final concentration of 0, 1or 5 μM/L, followed by incubated with PDGF-BB at a final concentration of 20 ng/ml. 1% DMSO were used as a solvent control. The U6 were used as a control gene. Data was expressed as Mean ± SD. *p < 0.05, **p < 0.01 vs. the normal control. ^#^ *p* < 0.05, ^##^ *p* < 0.01 vs. the PDGF-BB treated cells.

The plasma miR-638 levels were significantly decreased in PAH patients (*n* = 20) compared with control individuals (*n* = 30) (Figure 4A). A similar pattern was found, as shown by the significantly reduction in miR-638 expression levels in PDGF-BB-treated hPA-SMCs compared to the control cells, and the pre-treatment of the cells with lovastatin significantly re-gain the expression levels in miR-638. When the cells were treated with lovastatin at a high concentration of 5 μM, the miR-638 expression level were reversed to near the normal levels (Figure 4B), indicating that the expression levels of miR-638 were negative correlation to PAH both *in vivo* and *in vitro*.

## Discussion

In this study, we demonstrated several valuable findings for the first time. Firstly, the expression levels of miR-638 were decreased in patients with PAH. Secondly, in patients with low-HDL-C levels, plasma levels of miR-638 were significantly increased after XZK treatment. Thirdly, lovastatin (one of the main ingredients of XZK) significantly re-gains the expression levels of miR-638 in PDGF-BB-treated hPA-SMCs.

Previous studies on miR-638 have focused on several types of cancers and pulmonary hypertension, the role of miR-638 in lipid metabolism has not been reported. In this study, we described the low expression of miR-638 in patients with low HDL-C for the first time. Recent studies have demonstrated that miR-638, which is highly expressed in human vascular smooth muscle cells (VSMCs), targets the orphan nuclear receptor NOR1 (a key regulator associated with proliferative vascular diseases) as a novel regulator to inhibit proliferation and migration (Li et al., 2013; Chen et al., 2019). These studies suggested that miR-638 may be a potential therapeutic target for the prevention and treatment of human vascular diseases, such as atherosclerosis and pulmonary hypertension (Luque et al., 2018; Mansueto et al., 2020; Zhang et al., 2020).

XZK is a traditional Chinese medicine with multiple cardioprotective effective, which contains isoflavones and lovastatin and unsaturated fatty acid. Studies have shown that the effect of XZK in reducing TC and LDL-C levels is equivalent to conventional doses statins, and increasing HDL-C levels (Xu et al., 2017; Jia et al., 2020). Our efficacy data were consistent with previous studies. In the present study, LDL-C decreased by 14.6% and HDL-C increased by 11.2% after treatment with XZK 1,200 mg daily for 6 months. In previous Chinese trials with XZK 1,200 mg daily, LDL-C decreased by approximately 17%, HDL-C concentration increased by 4.6% (Li et al., 2010). However, previous studies have not explored the epigenetic mechanism of XZK. We found that miR-638 changed after the treatment of XZK, which is synchronized and related to the changes of HDL-C, suggesting that XZK may have potential value in the treatment of pulmonary hypertension by regulating HDL-C metabolism and miR-638 expression. As recent reported (Wang et al., 2020), serum HDL-C is lower in PAH patients comparing with healthy people, suggesting that HDL-C would be a potential biomarker for prediction and assessment of PAH. In the present study, the miR-638 raising effect of XZK were found negatively correlated with baseline HDL-C levels, indicating that XZK may have a potential therapeutic effect in PAH patients with low HDL-C.

Furthermore, *in vitro* experiments found that expression of miR-638 was increased in PDGF-BB-treated hPA-SMCs by lovastatin, one of the main ingredients of XZK, indicating that the miR-638-raising effect of XZK is at least partially related to lovastatin. However, the therapeutic effect of XZK cannot be explained by lovastatin alone. Besides lovastatin, XZK also contains other ingredients, including statin homologue, a variety of essential amino acids, unsaturated fatty acid, sterol, and small amounts of flavonoids. Whether XZK can be a potential drug for the treatment of pulmonary hypertension needs further *in vitro* and *in vivo* experiments and clinical investigations.

Although the mechanism of miR-638 in lipid metabolism and PAH is still unclear, previous studies have shown that circulating miR-638 may lead to the disorder of HDL-C metabolism by regulating the gene expression pathway related to oxidative stress response. The recent study has uncovered MAFB is a potential target of miR-638, ectopic expression of MAFB strongly induced the expression of ABCG1 and ABCA1, the key mediators of cholesterol efflux, and then affected lipid metabolism (Yang et al., 2015; Kim, 2017). Another recent study demonstrated that miR-638 induced apoptosis through regulating STARD10 signaling, the expression of miR-638 was inversely correlated with the expression of STARD10 mRNA (Zhao et al., 2017). Several previous studies have shown that apoptosis play a vital role in pulmonary arterial hypertension (Lv et al., 2021; Zimmer et al., 2021), indicating that the association of miR-638 with PAH may be partially related to STARD10, a target gene of miR-638.

### Study Limitations

The present study had several limitations. First, since the primary purpose of XZK intervention study was only to observe the lipid-lowering effect of XZK, several parameters associated with pulmonary hypertension, such as the right heart and pulmonary artery pressure, were not measured. Future research should focus on higher-quality and more rigorous larger sample trials to validate the findings and hypothesis of this study. Second, whether there are “cross-talk” among pulmonary hypertension, miR-638 and HDL-C metabolism, and the mechanism in this cross-talk need be further revealed in subsequent studies.

## Conclusion

In summary, the present study identified that, in patients with low HDL-C levels, XZK therapy raised the expression of miR-638, the changes of miR-638 were negatively correlated with baseline HDL-C levels. The potential therapeutic effect of XZK in PAH patients with low serum HDL-C levels deserves further exploration.

## Data Availability

The raw data supporting the conclusion of this article will be made available by the authors, without undue reservation.
